# Complete Genome Sequence of Bacillus frigoritolerans JHS1

**DOI:** 10.1128/mra.00184-22

**Published:** 2022-05-24

**Authors:** Jun Hyeuk Shin, Mechthild Bömeke, Anja Poehlein, Jacqueline Hollensteiner

**Affiliations:** a Genomic and Applied Microbiology and Göttingen Genomics Laboratory, Institute of Microbiology and Genetics, Georg-August University of Göttingen, Göttingen, Germany; SIPBS, University of Strathclyde

## Abstract

Bacillus frigoritolerans JHS1 was isolated from the soil of a tomato plant (Solanum lycopersicum). The genome consists of one circular chromosome (5,552,463 bp) and a plasmid (16,118 bp) with an overall GC content of 40.57%. Using TYGS for taxonomic classification, strain JHS1 was assigned to the species *Bacillus frigoritolerans*.

## ANNOUNCEMENT

**FIG 1:**
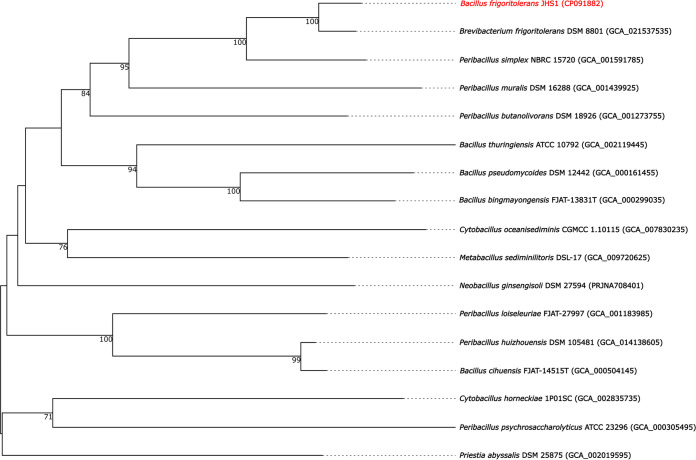
Phylogenetic classification of Bacillus frigoritolerans JHS1. The Genome BLAST Distance Phylogeny (GBDP) tree was generated using the Type Strain Genome Server (TYGS [18]; accessed 26 January 2022). The tree was inferred using FastME v2.1.6.1 (19) from GBDP distances calculated from the whole-genome sequences. The branch lengths are scaled in terms of GBDP distance formula d5. The numbers above the branches are the GBDP pseudo-bootstrap support values greater than 60% out of 100 replications, with an average branch support of 75.6%. The tree was rooted at the midpoint (20).

Bacillus frigoritolerans was first described in 1967 and isolated from dry soils in Morocco (1, 2). This species has potential for plant growth promotion by suppressing the growth of plant pathogens or stimulating seed growth (3–7). The rationale for sequencing strain JHS1 is to screen the genome for novel antifungal substances. Topsoil (5 g) was obtained from the rhizosphere of tomato plants in Göttingen, Germany (51.5197 N, 9.938 E), and heated for 5 h at 80°C (8, 9) to enrich for spore formers. Next, 1 g soil was mixed into 10 mL normal saline (0.85%); 1 mL was heated at 80°C for 12 min and cooled for 5 min on ice. A 10^−1^ dilution was plated onto medium 1 (DSMZ; 0.5% [wt/vol] peptone, 0.3% [wt/vol] meat extract, 1.5% [wt/vol] agar) and incubated at 30°C overnight. A colony was picked, restreaked onto medium 1, and incubated as described. Again, a colony was picked, raised in 10 mL LB overnight at 37°C, and harvested at 8,500 rpm. A single genomic DNA extraction was performed using the MasterPure complete DNA purification kit (Epicentre, Madison, WI, USA) with an initial lysis step with lysozyme (10) (495,000 U; SERVA, Heidelberg, Germany). The DNA was sequenced using Illumina and Nanopore technologies. Illumina libraries were constructed applying the Nextera XT DNA sample preparation kit (Illumina, San Diego, CA, USA) and run on a MiSeq instrument with the reagent kit v3 (600 cycles, 2 × 300 bp). A Nanopore library was prepared from high-molecular-weight DNA with the ligation sequencing kit 1D (SQK-LSK109) and the native barcode expansion kit (EXP-NBD114; barcode 17) (Oxford Nanopore Technologies, Oxford, UK). The MinION Mk1B device, with a R9.4.1 SpotON flow cell and MinKNOW software v21.10.4, was used for sequencing (72 h; Oxford Nanopore Technologies). Demultiplexing and base calling were performed using Guppy v6.0.1 (Oxford Nanopore Technologies) in high accuracy mode. Default parameters were used for all software unless otherwise specified. The Illumina and Oxford Nanopore sequencing resulted in 2,488,664 and 1,317,361 reads (*N*_50_/*N*_90_, 4,869 bp/1,441 bp), respectively. Quality filtering was performed using fastp v0.23.1 (11) and Porechop v0.2.4 (https://github.com/rrwick/Porechop.git; accessed January 2022). Unicycler v0.4.9 (12) was used to perform a hybrid assembly in normal mode, resulting in a closed circular chromosome (5,552,463 bp) and a closed circular plasmid (16,118 bp), with an overall GC content of 40.57%. The quality was inspected using Bandage v0.8.1 (13). The coverages, calculated using Qualimap v2.22-r1101 (14) with Bowtie 2 v2.4.4 (15) and Minimap2 v2.22 (16), were 102-fold (Illumina) and 650-fold (Nanopore), respectively. Annotation was performed using the Prokaryotic Genome Annotation Pipeline (PGAP) v6.0 (17). *Bacillus frigoritolerans* JHS1 harbors 5,190 predicted protein coding sequences (chromosome, 5,177; plasmid, 13). In addition, 42 rRNA, 52 regulatory RNA, 5 noncoding RNA (ncRNA), 1 transfer-messenger RNA (tmRNA), and 83 tRNA genes were detected. Based on whole-genome comparisons using TYGS (18), strain JHS1 was assigned to the species Brevibacterium frigoritolerans, with a digital DNA-DNA hybridization (dDDH-d4) value of 79.5% (confidence interval, 76.6 to 82.2%). The GC content differed minimally (0.06%) from the type strain Brevibacterium frigoritolerans DSM 8801 (GenBank accession number GCF_021537535), which supports the assignment (Fig. 1).

### Data availability.

The complete genome sequence is available at DDBJ/ENA/GenBank under the accession numbers CP091882 to CP091883. The raw reads were deposited at the NCBI Sequence Read Archive (SRA) under the accession numbers SRR18686634 (Illumina) and SRR18686633 (Nanopore).
